# Capturing Daily Disease Experiences of Adolescents With Chronic Pain: mHealth-Mediated Symptom Tracking

**DOI:** 10.2196/11838

**Published:** 2019-01-17

**Authors:** Chitra Lalloo, Amos Hundert, Lauren Harris, Quynh Pham, Fiona Campbell, Jill Chorney, Bruce Dick, Mark Simmonds, Joseph Cafazzo, Jennifer Stinson

**Affiliations:** 1 iOUCH Pain Lab Department of Child Health Evaluative Sciences The Hospital for Sick Children Toronto, ON Canada; 2 Centre for Global eHealth Innovation Toronto General Hospital University Health Network Toronto, ON Canada; 3 Department of Anesthesia and Pain Medicine The Hospital for Sick Children Toronto, ON Canada; 4 Department of Anesthesia Pain Management & Perioperative Medicine Dalhousie University Halifax, NS Canada; 5 Department of Anesthesiology & Pain Medicine University of Alberta Edmonton, AB Canada; 6 Centre for Global eHealth Innovation Techna Institute University Health Network Toronto, ON Canada; 7 Department of Child Health Evaluative Sciences The Hospital for Sick Children Toronto, ON Canada

**Keywords:** adherence, adolescents, chronic pain, disease experience, feasibility, mHealth, self-report, smartphones, symptom monitoring, mobile phone

## Abstract

**Background:**

Chronic pain is a common problem in adolescents that can negatively impact all aspects of their health-related quality of life. The developmental period of adolescence represents a critical window of opportunity to optimize and solidify positive health behaviors and minimize future pain-related disability and impaired work productivity. This research focuses on the development and evaluation of a smartphone-based pain self-management app for adolescents with chronic pain.

**Objective:**

The objectives of this study were to characterize (1) the feasibility of deploying a mobile health (mHealth) app (*iCanCope*) to the personal smartphones of adolescent research participants; (2) adherence to daily symptom tracking over 55 consecutive days; (3) participant interaction with their symptom history; and (4) daily pain-related experiences of adolescents with chronic pain.

**Methods:**

We recruited adolescents aged 15-18 years from 3 Canadian pediatric tertiary care chronic pain clinics. Participants received standardized instructions to download the *iCanCope* app and use it once a day for 55 days. Detailed app analytics were captured at the user level. Adherence was operationally defined as per the relative proportion of completed symptom reports. Linear mixed models were used to examine the trajectories of daily symptom reporting.

**Results:**

We recruited 60 participants between March 2017 and April 2018. The mean age of the participants was 16.4 (SD 0.9) years, and 88% (53/60) of them were female. The app was deployed to 98% (59/60) devices. Among the 59 participants, adherence was as follows: low (4, 7%), low-moderate (14, 24%), high-moderate (16, 27%), and high (25, 42%). Most (49/59, 83%) participants chose to view their historical symptom trends. Participants reported pain intensity and pain-related symptoms of moderate severity, and these ratings tended to be stable over time.

**Conclusions:**

This study indicates that (1) the *iCanCope* app can be deployed to adolescents’ personal smartphones with high feasibility; (2) adolescents demonstrated moderate-to-high adherence over 55 days; (3) most participants chose to view their symptom history; and (4) adolescents with chronic pain experience stable symptomology of moderate severity.

**Trial Registration:**

ClinicalTrials.gov NCT02601755; https://clinicaltrials.gov/ct2/show/NCT02601755 (Archived by WebCite at http://www.webcitation.org/74F4SLnmc)

## Introduction

Chronic pain in adolescents is a common problem that can negatively impact all aspects of health-related quality of life [1,2]. A significant proportion of these adolescents continue to experience pain that persists into adulthood [3,4]. However, the developmental period of adolescence also represents a critical window of opportunity to optimize and solidify positive health behaviors and minimize future pain-related disability and impaired work productivity [5,6].

This research focuses on the development and evaluation of a smartphone-based pain self-management app (*iCanCope*) for adolescents with chronic pain. The *iCanCope* app was developed through a phased, user-centered design approach. In phase 1A, a qualitative needs assessment study was completed with a sample of adolescents with chronic pain (n=23; age 14-18 years) and health care providers (n=7) [7]. Participants took part in focus group or individual interviews to identify their self-management needs and how an app could be designed to meet these needs. In phase 1B, a scoping review was completed to identify and characterize publicly available “pain apps” [8]. In a systematic search of the Apple, Android, Windows, and BlackBerry stores, 279 apps were identified. However, no single app was comprehensive in terms of pain self-management content. In addition, only 8.2% of apps involved a health care professional in the development process; patient engagement was limited, and no apps provided a theoretical rationale. In phase 2A, group design sessions were held with end users (adolescents with chronic pain), app designers, and members of the research team. These design sessions were intended to better understand (1) a typical “day in the life” of a young person with pain; (2) the various points when pain interfered with their function; and (3) how a pain self-management app could be designed to fit into their life. In phase 2B, a prototype app was designed by a team of professional designers and human factors specialists. The prototype then underwent iterative cycles of usability testing with a sample of 15 young people with chronic pain to ensure that it was easy to use and perceived as valuable [9]. In phase 3, a pilot randomized controlled trial (RCT) was conducted to evaluate trial feasibility with a sample of 60 adolescents. It is recommended that electronic health (eHealth) evaluations should use reasonable comparison groups rather than no-treatment or usual care. Thus, participants were randomized to receive one of two possible versions of the *iCanCope* app. Versions A and B included an identical symptom reporting function called the “Check-in.” Version B included additional self-management content related to goal setting, pain coping, and social support. In the context of the pilot RCT, we sought to compare app user groups in terms of adherence to symptom tracking while offering all participants a similar study condition (ie, a smartphone app) and a comparable amount of attention from the study team. In phase 4 (future), the pilot RCT will be scaled up to a definitive trial to evaluate the effectiveness of the *iCanCope* program for improving health outcomes. In the context of the definitive RCT, we will compare app user groups in terms of health outcomes over time. These health outcome data will be captured via validated questionnaires to be administered at baseline, 2 month, and 6 month timepoints.

The *iCanCope* symptom tracker applies the principles of ecological momentary assessment (EMA), which refers to a collection of methods that gather longitudinal, real-time data from individuals in their everyday environments [10]. Use of EMA has been shown to improve data quality by minimizing the potential biases associated with retrospective self-report data (eg, memory and self-concept biases) [10,11]. Mobile administration of EMA on devices such as smartphones can markedly improve patient adherence with daily diary reports compared with paper-based approaches [12]. It can also facilitate the capture of time- and date-stamped data, provide users with multiple response options, and embed branching logic for survey questions [12,13]. Thus, EMA can provide a data-rich window into the daily experiences of individuals across a variety of backgrounds and settings. While classical EMA studies are designed to collect dense, contemporaneous data for research use, the purpose of the *iCanCope* symptom tracker is to empower adolescents to track their symptoms, visualize trends, and communicate this information with people of their choosing (eg, caregivers, health care providers).

In a systematic review of pediatric studies that applied mobile-based EMA methodologies, Heron et al [13] identified 24 unique studies published from inception to May 2016 and found that EMA can be successfully implemented with children as young as age 7. In addition, they identified gaps in the existing literature to be addressed in future pediatric EMA studies. Specifically, they recommended that (1) researchers should evaluate the feasibility of youth using their own smartphones to participate in EMA studies; (2) EMA methods should be used to obtain a more complete picture of youth’s daily experiences with chronic medical conditions, including disease-related symptoms; and (3) self-report pediatric EMA measures should use pictorial response options instead of traditional Likert scales to optimize comprehension and engagement.

To begin addressing these identified knowledge gaps, this paper focuses on the symptom tracking data from the *iCanCope* phase 3 pilot RCT. Data related to intervention effectiveness will be published separately once the phase 4 trial is complete. The specific research questions (RQs) to be addressed in this paper are as follows:

RQ1: Is it feasible for a symptom-monitoring app to be remotely deployed to the personal smartphones (iOS or Android) of adolescent research participants?

RQ2: How adherent are 15-18-year olds with chronic pain to a regimen of daily symptom tracking with automated reminders over 55 consecutive days?

RQ3: Over a period of 55 days, how often do 15-18-year olds with chronic pain choose to view their history of self-report symptom data?

RQ4: What are the daily pain-related experiences of 15-18-year olds with chronic pain as per their self-report of pain intensity, pain interference, mood, physical activity, sleep quality, and energy over 55 days?

## Methods

This study was approved by the locally responsible Research Ethics Boards. A 2-arm, parallel-group RCT design with 1:1 group allocation was used (Multimedia Appendix 1) [14]. As per recommendations for feasibility studies, a sample size of 20-30 participants per group was targeted [15]. Adolescents were recruited from 3 pediatric tertiary care chronic pain clinics across Canada. Individuals were eligible if they were aged 15-18 years, were diagnosed with chronic pain, were English speaking, and owned a compatible smartphone (ie, iPhone 5 or later or Android device running operating system 4.4.2 or later). Chronic pain was defined as pain that had persisted or recurred for at least 3 months [5]. Individuals were excluded if they had moderate-to-severe cognitive impairment as per their health care provider. Participants were randomized to use one of two possible versions of the *iCanCope* app for a period of 55 days. Versions A and B included an identical symptom reporting function called the “Check-in.” Version B also included content related to goal setting, pain coping, and social support. The *iCanCope* app was downloadable from the Canadian Google Play (Android) or App Store (iOS). Each participant completed a guided orientation with research staff to download and learn how to use the app. This orientation was completed over Skype or over the telephone and took approximately 10-15 minutes. During orientation, participants received standardized instructions to download the *iCanCope* app onto their personal device and to sign-in with unique log-in credentials. Participants were asked to complete a symptom Check-in and access the History feature during orientation. These data (ie, first Check-in and History access) were excluded from all analyses because they were created by participants under the direction of research staff, rather than independently. In addition, participants were shown how to customize the time of the daily Check-in notification. Successful deployment was operationally defined as a participant downloading the app, logging in, and setting up his or her app profile.

**Figure 1:**
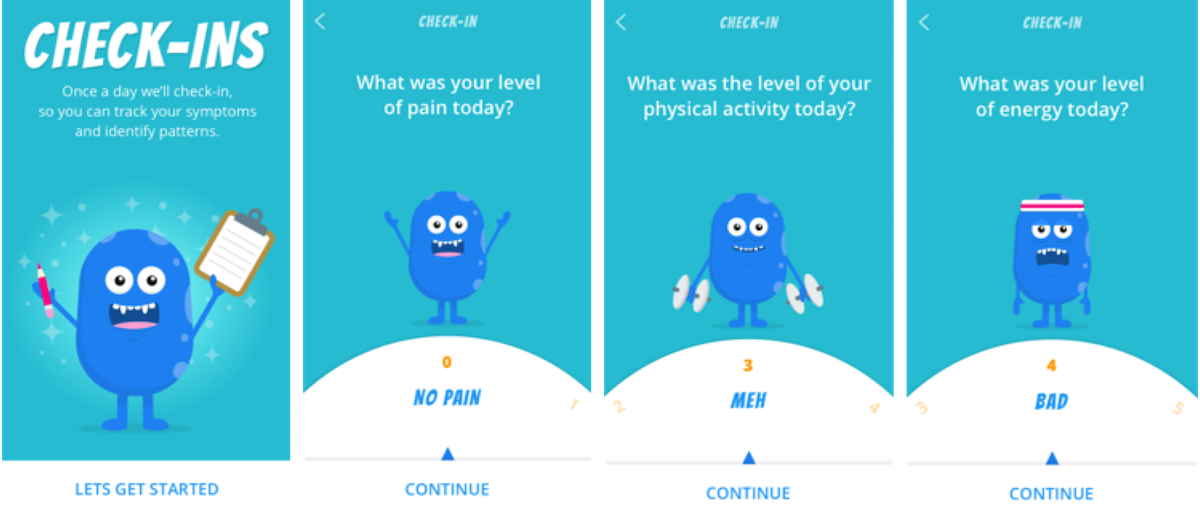
Example screenshots of the *iCanCope* daily symptom Check-in. From left: introductory screen; lowest anchor of pain intensity scale; mid-anchor of physical; activity scale; high-anchor of energy scale.

Participants were instructed to complete one symptom Check-in per day for a period of 55-days following their orientation. The 55-day study period was chosen on the basis of precedent from other Web-based self-management programs for adolescents with chronic pain, which found this duration to be associated with acceptable program adherence and effectiveness [16]. The daily Check-in feature used adolescent-friendly language and pictorial response options to optimize participant engagement (Figure 1). The pain intensity was self-reported on a 0-10 numerical rating scale with the anchors “no pain” and “worst pain.” Other symptom categories (pain interference, mood, physical activity, sleep quality, and energy) were captured via individual 5-point scales where a lower score indicated better function. Participants received daily push notification reminders at a time of their choice. Each study participant received a Can $15 gift card in recognition of their time and effort. In addition, each participant received a gift card valued at Can $40 as compensation for using their personal smartphone and data plan during the study.

**Figure 2:**
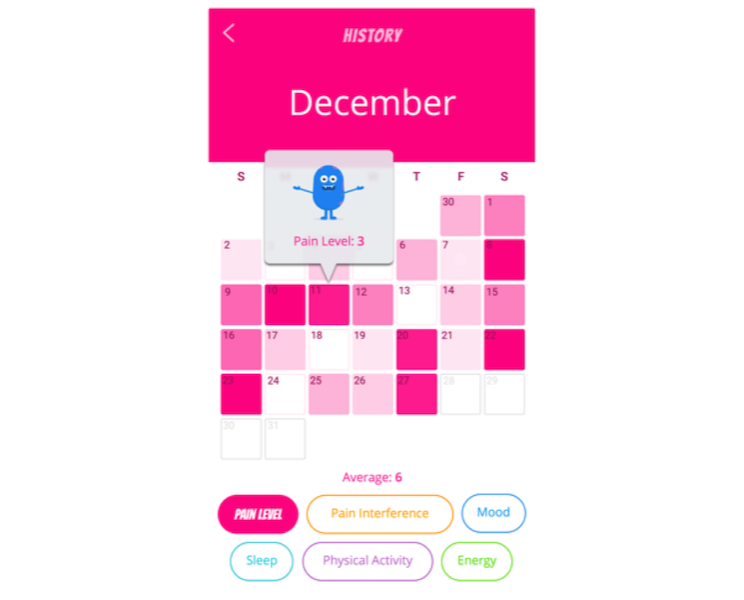
Example screenshot of the *iCanCope* History feature.

Participants could access the History function within the app at any time (Figure 2). This function allowed participants to view all of their previous Check-in data. The interface was designed as an dynamic calendar with a transposed “heat map” where different colors correspond to different symptoms (eg, pain intensity). The app is designed such that when the users open the History section, they are shown the pain intensity-specific heat map by default. Each day on the monthly calendar represented a potential Check-in day. If a participant had completed a Check-in on a particular day, that calendar day would be filled with color. More severe ratings were denoted by a darker color shade, which enabled users to examine patterns at the macro calendar-month level. Participants could click on any colored box within the calendar to view their exact numerical rating for that symptom. Furthermore, they could switch between different symptom categories using central filter buttons. The home screen of the app featured a central “banner” that displayed revolving messages to the user, such as “welcome home.” Users could swipe on the banner to generate a new message. A reminder related to the History function was one of the revolving banner messages periodically displayed to all users. However, participants did not receive any push notification reminders specific for the History function.

Detailed app usage analytics were captured at the individual level. Stata Version 15 (StataCorp LLC) software was used for all analyses [17]. The study team was equipped to centrally track any technical issues encountered during app deployment. User adherence was operationally defined as the relative proportion of symptom Check-ins that were completed over the 55-day study period: “low adherence,”≤24% (<13/55 reports); “low-moderate adherence,” 25%-49% (14/55 to 27/55 reports); “high-moderate adherence,” 50%-75% (28/55 to 41/55 reports); “high adherence,” 76%-100% (42/55 to 55/55 reports). Descriptive statistics were used to summarize the data for RQs 1-3, broken down by an assigned version of the *iCanCope* app. These data were analyzed to assess measures of central tendency (mean, median) and dispersion (SD, interquartile range). For RQ4, linear mixed models using an independent covariance structure and allowing for random slope and intercept were used to separately examine trajectories of each daily symptom over 55 days [18]. The estimated overall and user-level regression lines over the study period were plotted.

## Results

### Participants

This study was approved by the locally responsible Research Ethics Boards. A sample of 60 adolescents was recruited between March 13, 2017, and April 4, 2018; participants were recruited from clinics in Edmonton, Alberta (18, 30%); Halifax, Nova Scotia (15, 25%); and Toronto, Ontario (27, 45%). The mean age of participants was 16.4 (SD 0.9) years, and 88% (55/60) participants were female. Of the 60 participants, the majority (45, 75%) were iPhone users, with the remainder (15, 25%) being Android users; the breakdown of the *iCanCope* app assignment was as follows: version A (28, 47%) and version B (32, 53%). Table 1 shows additional participant demographic information.

### Research Question 1: Feasibility of the App Deployment

No technical issues were encountered during deployment. The *iCanCope* app was successfully deployed to 98% (59/60) devices. The single participant who did not receive the app completed the telephone orientation. However, this participant did not complete the steps required for app setup and also failed to log-in throughout the study. No technical issues were noted in this case. The analytics data presented for RQs 2-4 are drawn from 59 participants.

### Research Question 2: Participant Adherence to Regimen of Daily Symptom Tracking

**Figure 3:**
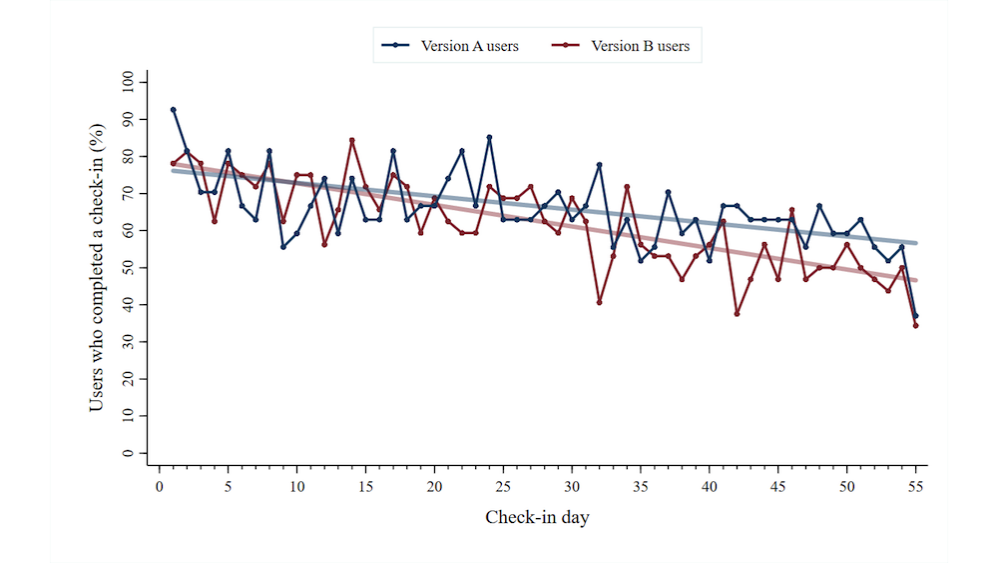
Total number of users who completed a symptom Check-in as a function of time (N=59).

The mean number of completed daily Check-ins was 36.0 (SD 13.9) for version A participants and 33.8 (SD 13.6) for version B participants. As per the operational definitions of adherence, version A participants (n=27) were distributed as follows: low (2, 7%), low-moderate (5, 19%), high-moderate (7, 26%), and high (13, 48%). Version B participants (n=32) were distributed as follows: low (2, 6%), low-moderate (9, 28%), high-moderate (9, 28%), and high (12, 38%). Figure 3 displays the total number of users who completed a Check-in as a function of time, broken down by the app version.

### Research Question 3: Participant Interaction With History of Symptom Check-in Data

**Figure 4:**
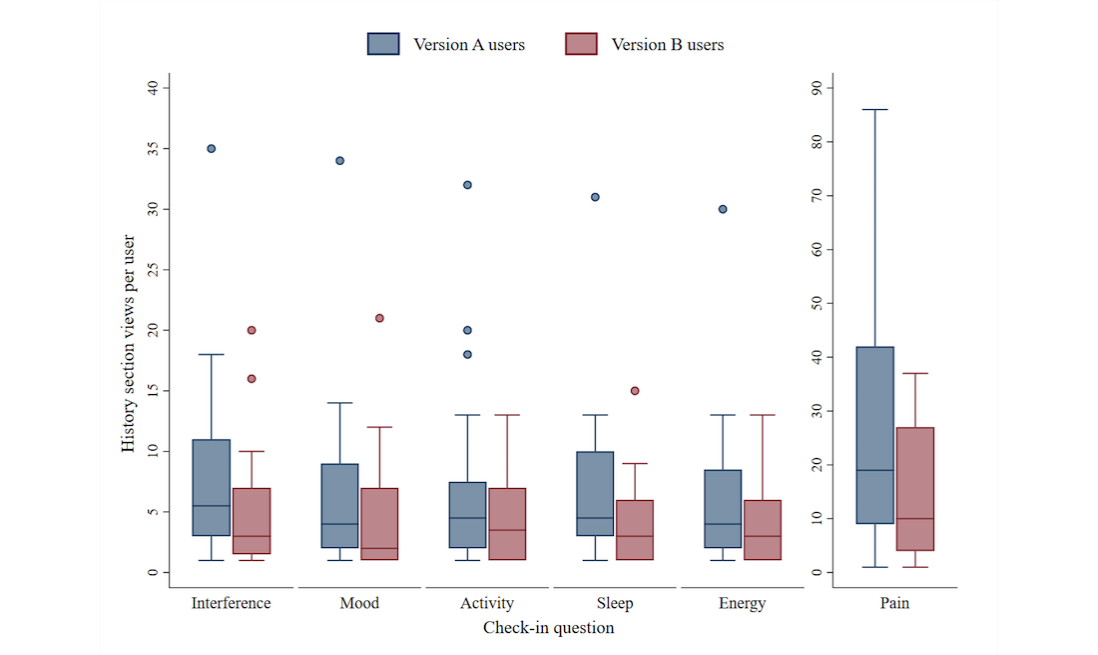
Interaction with the *iCanCope* History function to view symptom trends. The central line within each box denotes the median view count; the lower and upper box hinges denote the 25th and 75th percentiles, respectively; adjacent lines of the whiskers represent the lower and upper adjacent values, respectively; data points above each box represent outlier values.

Overall, 83% (49/59) participants accessed the History function at least once during the 55-day study period. Figure 4 displays the breakdown of views for each symptom category within History according to assigned the app version.

The app is designed such that when users open the History section, they are shown the pain intensity heat map by default. The total view count includes users who opened the History section multiple times in the same day or filtered between different symptoms within the same viewing session.

**Table 1:** Demographic and chronic pain characteristics of the study sample (N=60).

Characteristic		Participants, n (%)
**Age (years)**		
	15	11 (18)
	16	23 (38)
	17	18 (30)
	18	8 (13)
**Type of pain^a^**		
	Abdominal	17 (28)
	Facial	6 (10)
	Headache	24 (40)
	Low back	22 (37)
	Musculoskeletal	18 (30)
	Neuropathic	11 (18)
	Pelvic	10 (17)
	Other	20 (33)
**Duration of pain (years)**		
	<1	2 (3)
	1-5	32 (54)
	≥5	24 (40)
	Missing data	2 (3)

^a^Participants were able to report more than one type of pain.

### Research Question 4: Daily Pain-Related Experiences of 15-18-Year Olds With Chronic Pain

**Figure 5:**
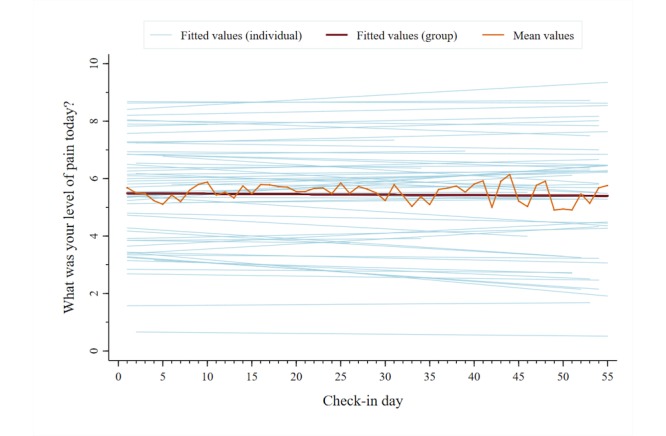
Self-reported pain intensity across 55 days using the *iCanCope* daily Check-in.

**Figure 6:**
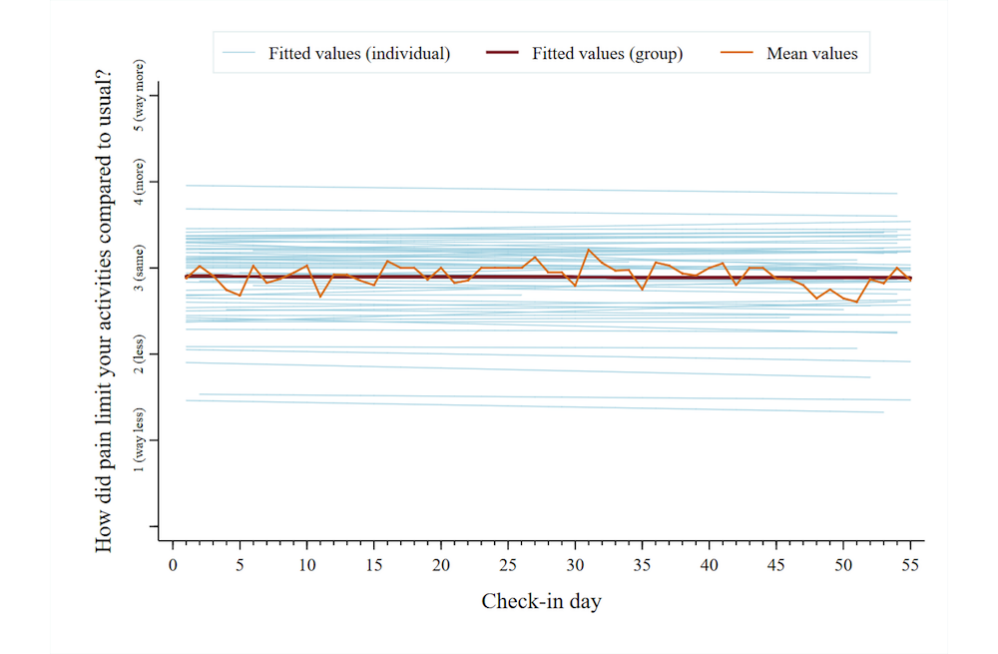
Self-reported pain interference across 55 days using the *iCanCope* daily Check-in.

**Figure 7:**
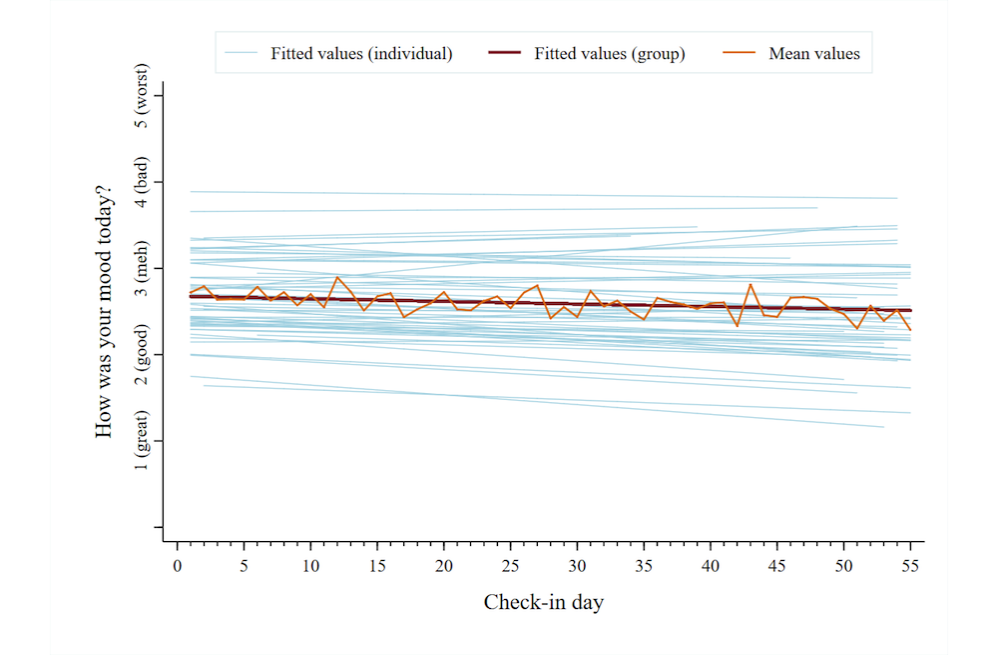
Self-reported mood across 55 days using the *iCanCope* daily Check-in.

**Figure 8:**
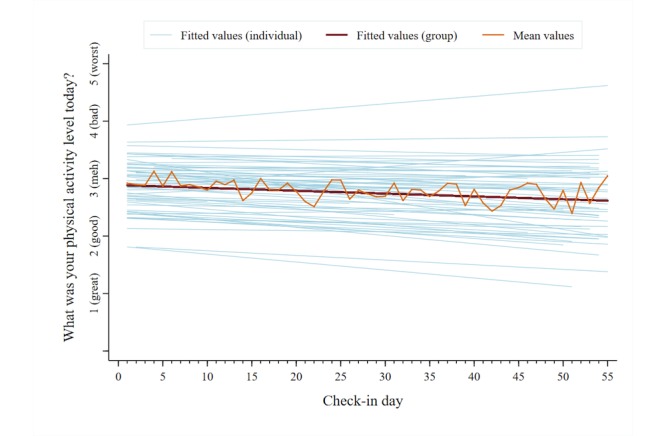
Self-reported physical activity across 55 days using the *iCanCope* daily Check-in.

**Figure 9:**
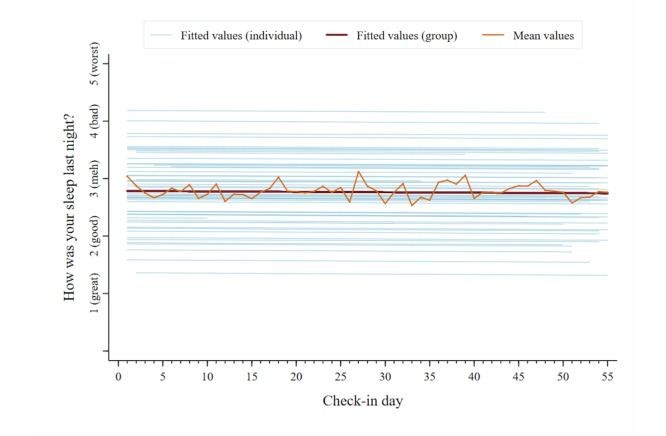
Self-reported sleep quality across 55 days using the *iCanCope* daily Check-in.

**Figure 10:**
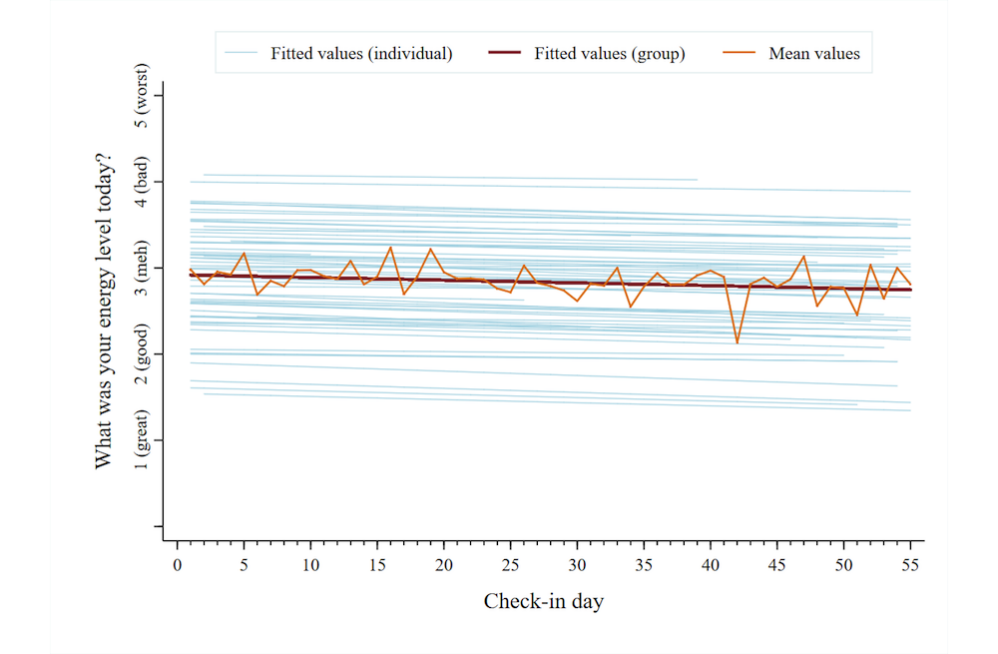
Self-reported energy across 55 days using the *iCanCope* daily Check-in.

A total of 2053 unique data points were analyzed for each symptom category across users. The mean reported pain intensity, captured on a 0-10 numerical rating scale, was 5.5 (SD 2.4). The mean scores for the other symptom categories, captured on individual 1-5 pictorial Likert scales, were as follows: pain interference, 2.9 (SD 1.0); mood, 2.6 (SD 1.0); physical activity, 2.8 (SD 1.1); sleep quality, 2.8 (SD 1.1); and energy, 2.9 (SD 1.0). Figures 5-10 present trajectories of each daily symptom over the 55 days.

## Discussion

### Principal Findings

Our data demonstrate that a symptom-monitoring app can be remotely deployed to the personal smartphones of adolescents using the infrastructure of the public app stores with a high degree of feasibility (98%, 59/60). The majority of participants exhibited either “high-moderate” or “high” adherence to a regimen of daily symptom tracking. Most (49/59, 83%) participants chose to view and interact with their symptom data through the History function. On average, participants who received version A of the app were more engaged with the symptom tracking feature than those who received version B. Adolescents with chronic pain reported pain intensity and pain-related symptoms of moderate severity, and these reports of their daily disease experience tended to be stable over 55 days.

### Comparison With Previous Work

As per the 2 existing systematic reviews of pediatric EMA research, no single study has focused on the daily disease experiences of adolescents with chronic pain [13,19]. However, one EMA study focused on the symptomology of youth with juvenile idiopathic arthritis, a condition that is associated with persistent pain [20]. In a sample of 59 individuals (aged 8-18 years) with arthritis, participants completed a mobile EMA protocol with a sampling frequency of 3 times daily over 28 days. The mean self-reported pain intensity was 36 (SD 23) on a 0-100 scale, which was characterized in the mild-to-moderate range. A subgroup of children (13/59, 22%) reported pain intensity in the high range (>40 of 100) [20]. In a 5-year retrospective study of 2249 patients presenting at a tertiary care pediatric pain clinic in Germany, the sample was characterized by moderate-to-high functional impairment and a mean recalled pain intensity of 6.4 (SD 2.1) over the past 4 weeks [21]. In this study of Canadian adolescents from a tertiary care chronic pain setting, participants reported a mean pain intensity of 5.5 (SD 2.4) and pain-related symptoms of moderate severity that tended to be stable over time. These comparisons suggest that our group of participants is similar to the German sample in terms of chronic pain intensity and impact on the function.

### Considerations for Future Pediatric Mobile Ecological Momentary Assessment Studies

#### Study-Issued Phone Versus Personal Phone

In the systematic review by Heron et al, 11 (46%) studies used smartphones for EMA administration [13]. In all of these studies, participants were issued a smartphone for the study duration rather than being required to download the required software onto their own device. In addition, most studies took steps to “lock down” the study devices by limiting their technical capabilities, such as blocking users from accessing other apps or disabling the phone function. This approach offers researchers with a high level of control over the EMA deployment process and the manner in which participants can interact with the study device. A potential disadvantage of requiring participants to carry a secondary device is that it may disrupt their typical routine (“ecology”) and potentially influence their reports. In this study, we chose to deploy the *iCanCope* app to the personal smartphones of study participants. While ceding some control over the deployment process, this approach was intended to encourage adolescents to incorporate the app into their daily routine, including their smartphone-related habits. Given the high penetration of mobile technology in this age group, we also sought to avoid the inconvenience of participants being required to carry multiple devices (personal and study-issued) for 55 days. Indeed, recent pediatric mobile health (mHealth) studies have cautioned against the use of secondary devices, as participants frequently left their study-issued device at home and, thus, missed report notifications [22,23].

#### Deployment Strategies and Future Scalability

We chose to use the existing infrastructure of publicly accessible app stores (iOS, Android) rather than a mobile device management system (eg, *MobileIron*, *AirWatch*) due to the lower burden for study participants and greater potential for scalability once *iCanCope* is publicly released. By carefully codifying the process of deployment, including both electronic manuals and telephone support from research staff, we were able to install the app onto participant devices with a high success rate. Upon public release of *iCanCope*, we anticipate that app deployment will be remotely supported through Web-based manuals, instructional videos, and email technical support, rather than the individualized telephone orientations used in the pilot RCT. We will apply our operational definition of successful deployment (user downloading the app, logging in, and setting up his or her profile) to measure the effectiveness of these self-guided strategies compared with telephone orientation. In addition, we will monitor user engagement with the future public app compared with the app evaluated through the pilot RCT. Differences between these user groups will include access to monetary compensation (ie, honoraria for study participants only), direct contact with the research team for study participants only, and potential duration of usage (ie, 55 days for study participants vs unlimited access for public users).

#### Benchmarks for User Adherence

In a systematic review focused on pediatric adherence to mobile EMA protocols, Wen et al identified 42 unique studies that included participants from clinical (16, 38%) and nonclinical (26, 62%) settings [19]. Adherence was typically defined as the proportion of prompts to which participants responded. Among the clinical studies, the average adherence was significantly lower in studies that prompted participants 2-3 times (73.5%) or 4-5 times (66.9%) daily compared with studies with a higher sampling frequency (>6 times; 89.3%). Stone and Shiffman have recommended that researchers should aim to achieve EMA adherence rates of ≥80% [24]. However, as the *iCanCope* app aims to provide useful data to adolescents about their symptomology, rather than to collect research or clinical data, the threshold for “success” is less defined. For instance, if a particular patient experiences little or no change in their daily pain intensity, they may not perceive value in tracking it daily for 55 days. In comparison with most studies identified in the 2 recent pediatric systematic reviews, this study implemented a lower sampling frequency (once vs 2-9 times daily) over a longer sampling duration (55 days vs 2-42 days) [13,19]. The decision regarding sampling frequency was informed by the conceptualization of *iCanCope* as a program for adolescents and based on the recommendations of patient partners during phase 1. Specifically, these collaborators recommended that we minimize the daily report burden, while also allowing users to create additional *ad hoc* reports if they wished. The decision regarding sampling duration was a function of the phase 3 pilot RCT design.

#### Design Considerations for Pediatric Studies

Most (63%) pediatric-focused EMA studies have reported on specific design considerations for children, including the use of youthful survey language [13]. In keeping with this trend, the *iCanCope* app design was informed by several core principles, which were developed in collaboration with patient partners during phase 1: (1) *keep it simple*; (2) *help me support my life, not just my pain*; and (3) *a safe and friendly space for me.* Based on these principles, the app was designed to include adolescent-friendly language and pictorial response options on the Check-in (see Figure 1). The specific symptoms tracked by the app were also chosen on the basis of recommendations of adolescents with chronic pain. We posit that these user-informed design choices may have contributed to the moderate-to-high adherence observed in this study and recommend this approach for future pediatric EMA studies.

### Considerations for User Engagement With App Symptom Tracking

#### Different Versions of the iCanCope App

In this study, participants were randomly assigned to use one of the two versions of the *iCanCope* app. Group-level analysis of the daily Check-in completion illustrated that participants who received version A were more adherent than participants who received version B (see Figure 3). One possible reason for this observed difference is that version A participants received a simpler app that was focused on symptom tracking. In contrast, version B participants received a more complex app with additional self-management content. The presence of these extra features may have diverted the attention of some participants away from the symptom tracking function. It is important to note, however, that the pilot RCT group sizes (n=28 for version A and n=32 for and version B) are limited for discerning the importance of this observed trend. The future phase 4 RCT will generate a larger pool of data to more definitively examine whether there are meaningful differences in symptom tracking adherence between the groups.

#### Access to Historical Symptom Tracking Data

Given that most EMA studies feature a high sampling density, participants are not typically granted access to their submitted reports due to the complexity of aggregating large volumes of data into digestible output in near real time. However, as *iCanCope* collects a manageable volume of data and is meant to empower adolescents, it was important to provide users with the ability to view their symptom trends. In general, participants accessed History multiple times over the course of the study, suggesting that they found value in this feature. During the phase 1 studies, adolescents indicated interest in using the History function to communicate with their health care team during clinic appointments [9]. This user requirement was taken into consideration when designing the History feature. For instance, a calendar interface was chosen so that users could access a bird’s-eye view of their symptom trends in response to common clinician queries about their pain and function since the last clinic visit. During app orientation, participants were shown how to use their History to communicate symptoms with their health care providers. Conceivably, some study participants did choose to use their data in this way during the study, although the research team did not track specific modes of use.

### Limitations

Some limitations of this study should be noted. The unique methodological characteristics of our study (eg, sampling density and duration, purpose of data collection) must be considered when making direct comparisons with traditional EMA studies. The low sampling density may have failed to capture daily fluctuations in pain and related symptoms. The app Check-in was the sole source of collected symptom data and was reliant on participant self-report. No additional symptom data sources were included such as wearable accelerometers or parent report. It was not feasible to track if and how participants chose to share their symptom History with their health care providers.

### Conclusions

This paper begins to address identified knowledge gaps in the field of adolescent EMA research through an mHealth app in pediatric chronic pain. We suggest that future research should extend our work by (1) evaluating the feasibility of deploying EMA apps to younger children; (2) experimenting with protocols of different sampling densities and durations; (3) triangulating self-report data with passive ambulatory data collection methods; and (4) examining other chronic disease groups.

## Appendix

Multimedia Appendix 1 CONSORT-EHEALTH checklist (V 1.6.2).

## Abbreviations

EMA: ecological momentary assessment
mHealth: mobile health
RCT: randomized controlled trial
